# How would you like your proximal femoral nail – with a monocortical recon plate, with cable, or neat? A functional and radiological study of reverse oblique (AO/OTA 31-A3) intertrochanteric femur fractures

**DOI:** 10.1051/sicotj/2024047

**Published:** 2024-12-03

**Authors:** Mehmet Süleyman Abul, Aytunç Metin, Ömer Faruk Sevim, Ömer Hekim, Engin Eceviz

**Affiliations:** 1 Department of Orthopedics and Traumatology, Kartal Dr. Lütf Kırdar City Hospital University of Health Sciences D-100 Güney Yanyol No:47 Cevizli 34865 Istanbul Turkey; 2 Department of Physiotherapy and Rehabilitation, Kartal Dr. Lütfi Kırdar City Hospital University of Health Sciences D-100 Güney Yanyol No:47 Cevizli 34865 Istanbul Turkey

**Keywords:** Reverse oblique intertrochanteric femur fractures, Proximal femoral nail, Cable augmentation, Monocortical recon plate

## Abstract

*Objective*: Intertrochanteric femur fractures (ITFF), more so reverse oblique fractures (AO/OTA 31-A3), are the most challenging clinically, with significant morbidity and mortality. Early stable fixation should be achieved to allow early mobilization and reduce complications. This study evaluates the functional and radiological outcomes of three Proximal Femoral Nail (PFN) techniques – PFN alone, Cable + PFN, and Monocortical reconstruction plate (MRP) + PFN– in managing reverse oblique ITFF, to determine the most ideal of them. *Methods*: A retrospective analysis was performed on 106 patients treated from 2015 to 2022. The patients were classified by the surgical intervention: Cable + PFN (*n* = 37), MRP + PFN (*n* = 29), and PFN (*n* = 40). The critical parameters analyzed included healing time, quality of reduction, rates of complications, and functional outcomes of Trendelenburg gait. *Results*: The bone healing time was significantly faster in the Cable + PFN group and MRP + PFN group as compared to the PFN group, 4.43 ± 0.92 and 4.44 ± 0.90 months versus 6.40 ± 2.41 months, respectively (*p* < 0.001). Compared with the PFN group, the number of cases with Trendelenburg gait in the Cable + PFN group was significantly lower, 10.8%. The number of patients showing the Trendelenburg gait trended lower in the MRP + PFN group but was insignificant (*p* = 0.075). Radiological outcomes did not differ significantly among the groups. *Conclusion*: The use of Cable + PFN and MRP + PFN techniques has superior outcomes with earlier bone union and far less incidence of Trendelenburg gait than PFN alone. These findings can help hint that perhaps the usage of cables and recon plates enhances the stability and functional restoration in patients who have sustained reverse oblique ITFF.

**Level of evidence**: III

## Introduction

**Figure 1:**
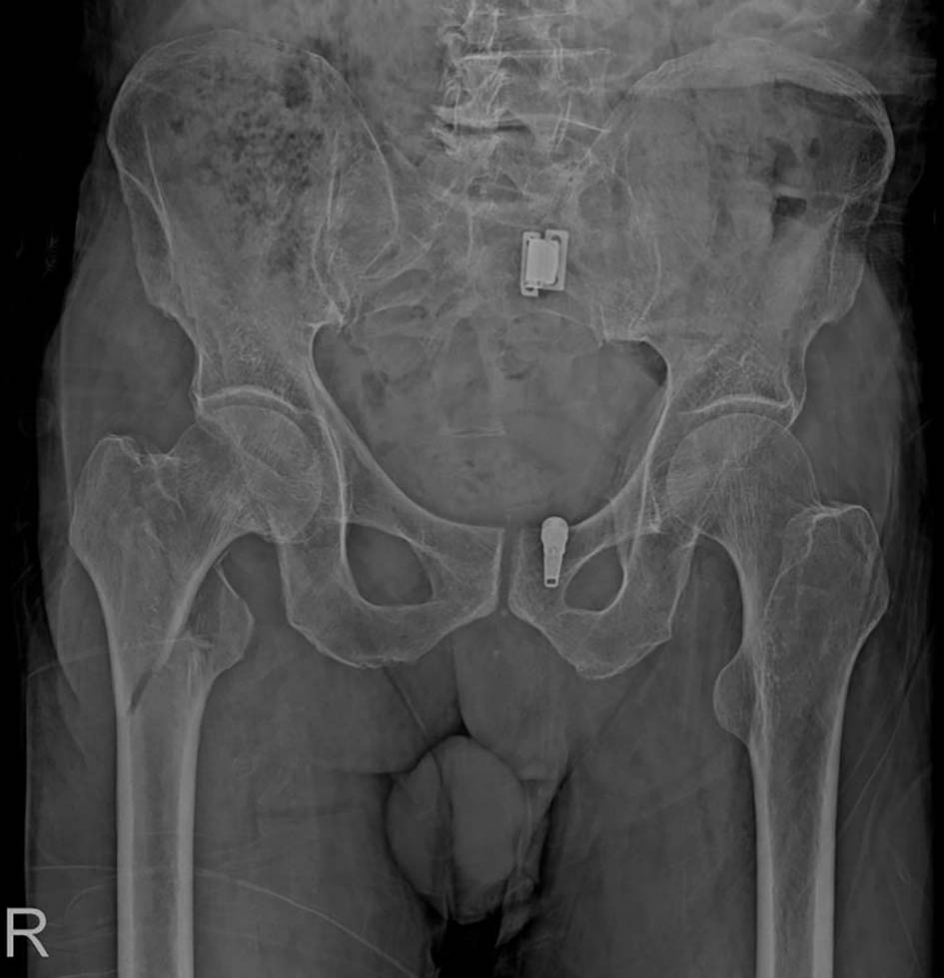
The pelvis and proximal femurs of one of the patients included in our study which demonstrates a typical AO/OTA 31-A3 fracture.

Intertrochanteric femur fractures (ITFF) are a significant clinical concern, more so in older people, given their high occurrence and the considerable morbidity and mortality associated with them. These fractures relate to the fractures that occur between the greater and lesser trochanters of the femur and may be of various types based on the fracture pattern, with reverse oblique intertrochanteric fractures (AO/OTA 31-A3) being one of the more problematic subtypes to deal with, hence the loss of lateral femoral cortical integrity [1–3]. These fractures exhibit an excess of obliquity in the fracture line, running in the reverse direction because of the counter forces of gluteus medius and iliopsoas muscles, which leads to difficulty in stabilization and healing [1–3] (Figure 1).

The main goal of treatment for reverse oblique intertrochanteric fractures is to provide stable fixation with immediate mobilization and early weight bearing to decrease the risks of complications such as nonunion, malunion, and prolonged immobility [3]. Various surgical techniques and fixation devices have been adopted in dealing with such fractures, including Proximal Femoral Nails (PFNs) [4–7]. Although quite commonly used, there still is a significant debate on the best surgical approach to these complicated fractures [8].

Other treatment techniques apart from PFN are the use of cables and plating [9, 10]. These alternative methods are hence considered because of the inherent limitations of the PFNs in managing reverse oblique intertrochanteric fractures. To counter the force of the gluteus medius muscle on the proximal fragment of the fracture, the addition of a cable and plate increases the stability of fixation [7], especially when used independently or in combination with a PFN. This may offer advantages, such as early healing [11] and improved ability for mobilization, or disadvantages, such as disruption of microcirculation and wound complications, depending on the specific clinical situation.

Solitary usage of proximal femoral plates offered superior biomechanical properties compared to the traditional PFN in the study of Polat et al. [12]. However, the use of monocortical reconstruction plates (MRP) in conjunction with PFN has not been explored as thoroughly. This paper will fill this gap by comparing, for the first time, the use of a MRP with PFN against the traditional combination of PFN and cerclage combination in the treatment of proximal femoral fractures.

**Figure 2:**
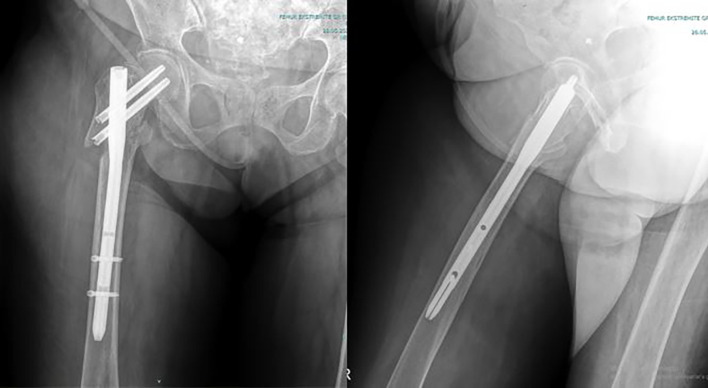
The postoperative radiographic appearance of a patient treated with a proximal femoral nail.

**Figure 3:**
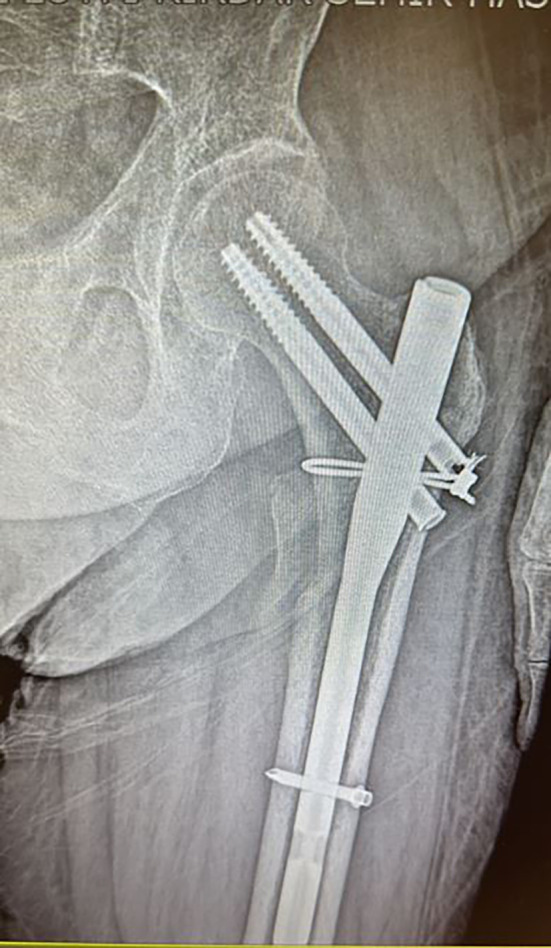
The postoperative radiographic appearance of a patient treated with a proximal femoral nail (PFN) combined with cerclage cable fixation.

**Figure 4:**
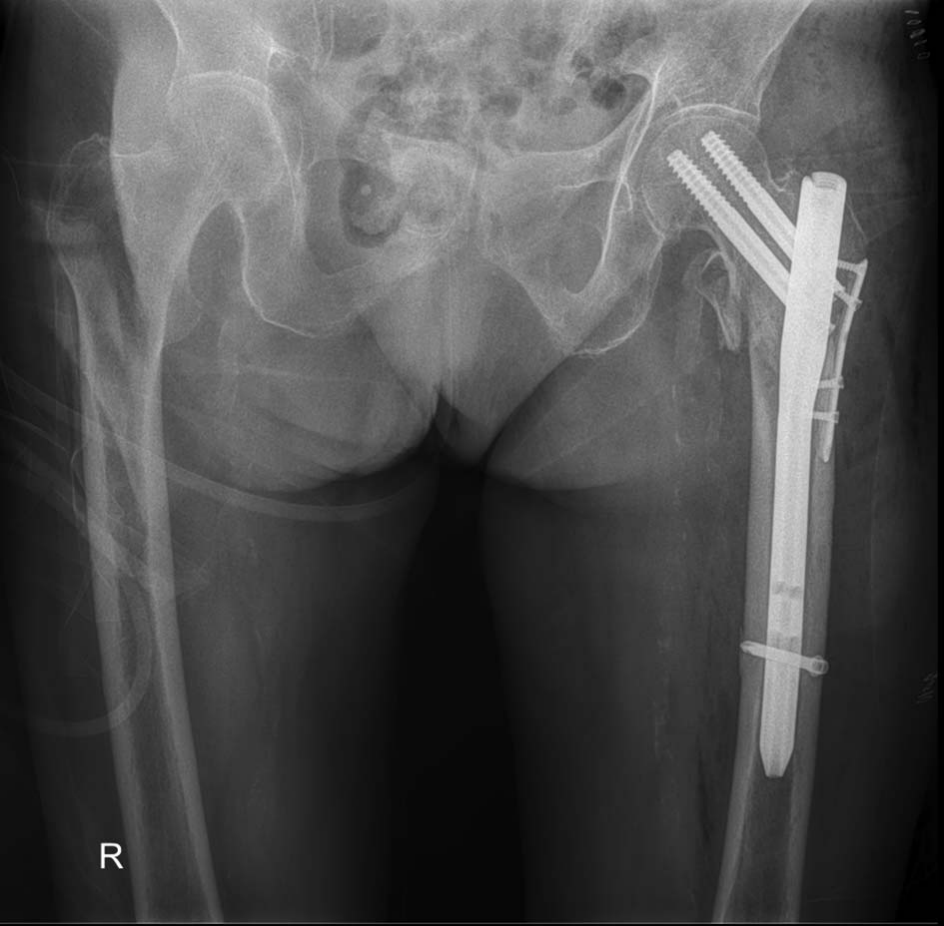
The postoperative radiographic appearance of a patient treated with a monocortical recon plate in combination with a proximal femoral nail.

The functional and radiological outcome of the present study was to evaluate the three different techniques in managing reverse oblique intertrochanteric fractures of the femur treated by PFN (Figure 2) and to compare it with Cable + PFN (Figure 3) and MRP + PFN (Figure 4). Based on the retrospectively analyzed data of the patients who were treated for seven years, we aim to determine the efficacy and safety of these surgical methods and to find out any difference that can contribute to a change in healing time, the incidence of complication rate, and functional outcome between the treatment groups.

**Figure 5:**
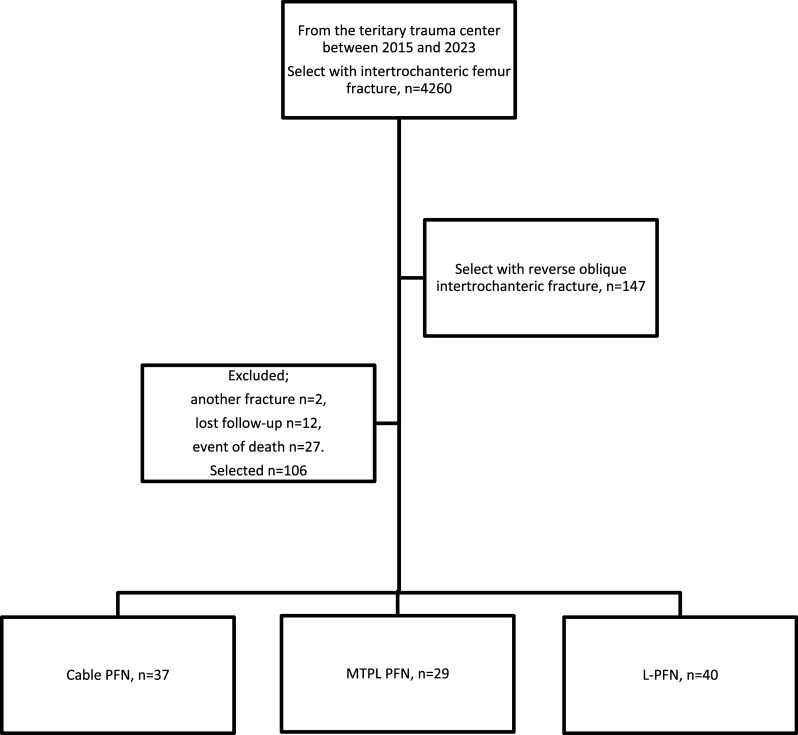
Flow chart.

## Methods

This retrospective study was conducted to assess the postoperative success and relative efficacy of various surgical techniques used to treat reverse oblique ITFF (AO/OTA 31-A3) at Kartal Dr. Lütfi Kırdar City Hospital from 2015 to 2022. The study sought to provide a comparative analysis of three surgical interventions: Cable + PFN(Cable + Profin^®^, TST), Monocortical Recon Plate PFN (3.5 mm Recon plate + Profin^®^, TST), and PFN (Profin^®^, TST). The adult and geriatric population of patients enrolled in the study consisted of those who underwent surgical intervention for ITFF. A total of 147 patients with reverse oblique ITFF were found initially. Exclusions included multiple fractures (*n* = 2), patients lost to follow-up (*n* = 12), and patients deceased during the interval (*n* = 27), making a total of 106 patients eligible for analysis (Figure 5). The final cohort consisted of 33 men and 73 women with a mean age of 74.17 ± 16.00 years (age 18–96). These patients were operated on by four qualified orthopedic surgeons who work in a third-level trauma center, and the choice of the implant was made by the surgeon’s decision.

The data were based on a few significant parameters that would ensure the findings of surgery procedures. Data on demographics, such as the age and gender of the patients, were recorded to assess potential impacts on surgical findings. The time for healing, which was the duration taken for the bone union after surgery, gave us the recovery period for each surgical method. Fracture reduction quality was labeled good, acceptable, or poor on postoperative radiographs following the modified Chang et al. [13] classification. Malunion and nonunion were noted to assess whether there was an achievement of adequate bone healing for each method classification [14]. Additionally, the presence of postoperative Trendelenburg gait, indicative of hip dysfunction, was recorded. The Presence of Trendelenburg gait was categorized as a contralateral pelvic drop of >4° during stance [11, 15]. Existing imaging studies and clinical examination findings of the affected extremity were reviewed to ensure comprehensive data analysis.

The inclusion criteria for this study were adult and geriatric patients who presented with ITFF, and the inclusion criteria stated that radiological and clinical follow-up data be complete. Patients with incomplete follow-up data or missing clinical information were not included. The retrospective nature of the study allowed us to complete this analysis over 3 months, which is adequate for reviewing data and drafting the manuscript. The data collection was performed through available imaging and patient information systems in the hospital by the principal and assistant researchers. This ensured that the clinical and radiological data were complete and accurate.

### Operation technique

The patient was positioned supine on a fracture table with traction applied to restore limb length and alignment. A small incision was made proximal to the greater trochanter, and entry into the femoral canal was achieved under fluoroscopic guidance using an awl or reamer. A guidewire was then advanced down the medullary canal, and sequential reaming was performed to accommodate the nail. The appropriate PFN was selected based on the patient’s anatomy and fracture pattern. The nail was inserted into the femoral canal, ensuring correct positioning under fluoroscopy. Proximal locking screws were placed through the nail to secure the femoral head, followed by distal locking screws to prevent rotational or axial instability. Fluoroscopic images confirmed the reduction and implant positioning before wound closure. Postoperative radiographs were taken to verify alignment and fixation. Some of the patients received only tip-entry proximal femoral nails, while others were treated with cable fixation to the proximal fragment junction after nail application. Additionally, some patients had fixation applied from the lateral side of the femur using two monocortical locking screws proximally and two distally of the fracture site.

All patients followed the same rehabilitation and analgesic protocol. Partial weight-bearing was initiated as soon as tolerated, and full weight-bearing was allowed only after sufficient healing was observed on X-rays.

## Results

In this retrospective case series, patients with ITFF fractures who presented at Kartal Dr. Lütfi Kırdar City Hospital between 2015 and 2022 were reviewed. A total of 4260 cases were identified with an ITFF fracture. Upon careful examination, 147 patients with reverse oblique intertrochanteric fractures were identified. Two had multiple fractures, and thus, they were excluded from the study, along with 12 who were lost to follow-up and 27 who died, which left 106 for analysis. Surgical interventions of these 106 patients were in 37 cases done by the application of Cable + PFN, in 29 cases done by the application of MRP + PFN, and in 40 cases done by the application of PFN. The mean age was 74.17 ± 16.00 years, without statistical difference between groups based on surgical intervention type (*p* = 0.954). Out of 106 cases, 73 were females (68.9%); no statistical relationship existed between groups for gender distribution (*p* = 0.376). The mean follow-up period for all groups was 53.8 ± 23.22 months, with no statistically significant difference between surgical groups (*p* = 0.503) (Table 1).

**Table 1:** Demographics.

Variables	Overall (*n* = 106)	Cable + PFN (*n* = 37)	MRP + PFN (*n* = 29)	PFN (*n* = 40)	*P* value
Age at admission (year)	74.17 (16.00)	74.16 (15.43)	73.79 (20.60)	74,47 (12.85)	0.954
Female, *n* (%)	73 (68.9%)	27 (73.0%)	17 (58.6%)	29 (72.5%)	0.376
Follow-up (months)	53.80 (23.22)	52.49 (23.29)	58.07 (18.86)	51.80 (26.03)	0.503

Radiologically, in the clinical outcomes of the groups, 53 cases (50.0%) showed good, 42 cases (39.6%) showed acceptable, and poor reductions in 11 cases (10.4%). There was no significant association between the type of surgery and the quality of reduction (*p* = 0.127) (Table 2). The mean time taken to heal the bones was 5.17 ± 1.89 months. Thus, healing times in the Cable + PFN and MRP + PFN groups were 4.43 ± 0.92 and 4.44 ± 0.90 months, respectively, while the PFN group healed for 6.40 ± 2.41 months. A significant difference from a statistical point of view was shown between the PFN group and all the others (*p* < 0.001) (Table 2).

**Table 2:** Clinical results.

Variables	Overall (*n* = 106)	Cable + PFN (G1) (*n* = 37)	MRP + PFN (G2) (*n* = 29)	PFN (G3) (*n* = 40)	*P-*value	*P-*value
						G1-G2
						G1-G3
						G2-G3
Reduction, *n* yes (%)						
Good	53 (50.0%)	24 (64.9%)	15 (51.7%)	14 (35.0%)	0.127	
Acceptable	42 (39.6%)	11 (29.7%)	11 (37.9%)	20 (50.0%)		
Poor	11 (10.4%)	2 (5.4%)	3 (10.3%)	6 (15.0%)		
Bone union (months)	5.17 (1.89)	4.43 (0.92)	4.44 (0.90)	6.40 (2.41)	0.000	1.000
						0.000
						0.000
Malunion, *n* yes (%)	18 (17.0%)	4 (10.8%)	3 (10.3%)	11 (27.5%)	0.080	
Nonunion, *n* yes (%)	9 (8.5%)	3 (8.1%)	2 (6.9%)	4 (10.0%)	0.896	
Trendelenburg, *n* yes (%)	21 (19.8%)	4 (10.8%)	4 (13.8%)	13 (32.5%)	0.037	0.500
						0.022
						0.075

Complications for the 106 cases included 18 (17.0%) malunion and 9 (8.5%) nonunions, with no significant difference between the surgical groups in the distribution of the two parameters (*p* > 0.05) (Table 2). Twenty-one patients complained of a Trendelenburg gait, which accounted for 19.8% of the whole study cohort. Analysis of the surgical groups revealed Trendelenburg gait in 4 cases (10.8%) of the Cable + PFN group, 4 cases (13.8%) of the MRP + PFN group, and 13 cases (32.5%) in the PFN group. There was a statistically significant relation between Cable + PFN and PFN groups about the development of Trendelenburg gait (*p* = 0.022). Although no statistical significance was noticed between the PFN and MRP + PFN groups, there was a trend favoring the PFN group in developing Trendelenburg gait (*p* = 0.075).

## Discussion

The most important finding of this study is the significant difference in bone healing times and the incidence of Trendelenburg gait among the three surgical techniques used to treat reverse oblique ITFF. The Cable + PFN and MRP + PFN groups showed superior outcomes compared to the solitary use of PFN. This important comparison may yield new insights and potential improvements in surgical outcomes for proximal femoral fractures. To better understand the reasons behind these findings, it is essential to examine each surgical instrument individually, considering their respective advantages and disadvantages on this specific fracture type.

PFN is widely regarded as an effective treatment option for reverse oblique intertrochanteric fractures due to its ability to provide strong biomechanical support and early mobilization. Studies such as those by Polat et al. and Thusoo et al. have demonstrated that PFN offers superior biomechanical properties, particularly in complex fracture patterns where stability is a major concern [12, 16]. The intramedullary placement of the PFN reduces the lever arm, thereby decreasing the risk of varus collapse and enabling earlier weight-bearing compared to extramedullary devices like dynamic hip screws (DHS) [17, 18]. However, PFN is not without its disadvantages. Complications such as screw cut-out, non-union, and implant failure have been reported, particularly in cases with reverse oblique ITFF [16, 18]. On that matter, enhancing the reduction technique with additional instruments like cable or MRP likewise in our study can be useful to prevent these complications.

Cerclage wiring, a straightforward and historical surgical technique, has been used in fracture fixation either alone or in combination with other implants [10, 11, 14, 15, 17, 18]. However, concerns about cerclage wiring include potential disruption to regional blood circulation, which could delay fracture healing [11, 14, 15]. Research on the cerclage-bone interface mechanics demonstrated that cerclage provided point contact fixation on the femoral shaft while preserving blood supply [15]. Clinically, numerous recent studies have confirmed the beneficial effects of cerclage wiring in managing peritrochanteric femoral fractures, as well as humeral shaft and acetabular fractures [10, 17]. It appears that cerclage wiring facilitates fracture reduction without hindering fracture healing as well as in our study.

Monocortical reconstruction plates (MRP) combined with Proximal Femoral Nails (PFN) have rarely been directly compared to other fixation techniques on patients in the existing literature, except in our study. A biomechanical study by Zhang et al. is the first and only study that specifically demonstrates the effectiveness of MRP as a viable alternative to cerclage in the treatment of unstable intertrochanteric fractures [19].

Their findings highlight several benefits of using MRP, including enhanced biomechanical stability, reduced implant stress, and decreased fracture displacement, which collectively contribute to better functional outcomes and lower complication rates [19]. As well as, the findings in our study that showed the MRP + PFN group, has superior results at healing time and gait performance of the patients. These advantages position MRP as a promising option for improving patient outcomes in cases where traditional methods might fall short.

In summary, the combination of PFN with MRP or Cable demonstrates superior outcomes with fewer complications related to bone healing, despite the increased cost associated with the procedure.

Radiological assessments classified the quality of fracture reduction as good in 50% of cases, acceptable in 39.6%, and poor in 10.4%. There was no significant difference in the quality of reduction across the three surgical techniques. This consistency effectively eliminates a confounding factor, allowing for a clearer understanding of the impact of implant choice. Nonetheless, it is important to acknowledge both the critical importance and the inherent difficulty of achieving optimal reduction in these fractures [20].

Our study observed a significant reduction in Trendelenburg gait in patients treated with Cable + PFN and MRP + PFN compared to those treated with PFN alone. This finding is consistent with the results of Imerci et al. [10] who reported that the use of cerclage cables provided additional mechanical support, leading to improved outcomes in patients with reverse oblique fractures. The mechanical support offered by the cables and the MRP likely contributed to the enhanced stability of the fixation by providing a counter-force against the gluteus medius muscle thereby reducing the incidence of Trendelenburg gait and associated complications. This alignment with the existing literature further validates the use of cable augmentation as an effective strategy to improve functional outcomes in patients with complex femoral fractures [10]. Although as noted by Sivakumar et al. [21] loss of femoral offset and the protrusion of lag screws can result in Trendelenburg gait. There is another possible cause of the disruption of the gait which is protrusion of the nail over time which is seen sole use of the PFN in the patients of the study by Sun Ju et al. [22]. To preserve the quality of the walk, we believe that using a nail in combination with MRP or Cable would be beneficial to maintain lateral wall support femur by resisting as a counter force to gluteus medius muscle. These findings could influence surgical decision-making, particularly in elderly patients where rapid mobilization and reduced complication rates are crucial for recovery.

Although this study gives vital information regarding the comparison of the performance of these fixation techniques in the present discourse on the optimization of treatment strategies for reverse oblique ITFF, it has several limitations that need to be mentioned at the outset. Its retrospective design includes inherent selection bias and limited control over a good number of confounding variables that may impact on the outcome. Though a high number of cases were identified in the initial survey, many exclusions for lost follow-up, multiple fractures, and patient deaths reduced the final sample size, potentially affecting generalizability. Generalization of practice and patient demographics from a single center may not be fully appropriate. The completeness and quality of clinical and radiological data are variable across the study period and the patient groups themselves, thus complicating robust comparisons between surgical techniques. Moreover, while the clinical and radiological outcomes were assessed quite comprehensively in the study, there was no detailed evaluation of patient-reported outcomes relating to functional status and quality of life, so further insights from this study are limited. With an average follow-up of about 54 months, fair judgment about medium-term outcomes can be made, but the long-term implications related to implant durability and late complications remain unexplored. Moreover, surgical techniques themselves or the sizes of implants or locking screws employed were left to the discretion of the surgeon or institutional practice and thus might have introduced bias into both treatment allocation and outcome assessment. Finally, there was a higher participation of older adults from a specific geographic region, thus generalizing findings to younger populations or other demographic groups. Addressing these limitations would give relatively full insight into the findings, underlining areas for future research in the optimization of treatment strategies for reverse oblique ITFF.

To minimize the selection, prospective, randomized controlled trial studies have to be done comparing the outcomes of different surgical techniques, including Cable + PFN, MRP + PFN, and PFN alone. Another important aspect would be the standardization of protocols for implant selection, including its type and size and type of locking screws, to allow more clarity on the effect of these variables on clinical and radiological outcomes. Incorporation of patient-reported outcomes in terms of functional recovery may also be very elaborately detailed in this study, giving room for the quality of assessment about the recovery process as well as the complication rates to have a more complete assessment of treatment effectiveness.

Secondly, it would be beneficial to concomitantly conduct multicenter studies with large and diverse patient populations to further strengthen external validity and the generalizability of findings. In addition, biomechanical studies on mechanical stability and long-term durability using different methods of fixation could be performed to help in developing evidence-based guidelines for treatment. By these lines of approach, future research can help in the optimization of surgical decision-making in order to improve patient outcomes with respect to reverse oblique ITFF.

## Conclusion

PFN remains a reliable option for treating reverse oblique ITFF, yet the incorporation of cerclage cables or MRP offers significant advantages in terms of faster healing and improved functional outcomes.The similar positive outcomes achieved with both the MRP and cerclage techniques indicate that MRP can be considered a promising alternative to cerclage.These findings may guide surgeons in optimizing treatment strategies to achieve better patient recovery and reduce complications associated with these challenging fractures.

## Data Availability

The datasets generated and/or analyzed during the current study are available from the corresponding author upon reasonable request.
